# Congenital absence of the inferior vena cava, a rare cause of deep vein thrombosis in adolescent: Case report

**DOI:** 10.1097/MD.0000000000043818

**Published:** 2025-08-08

**Authors:** Dongwon Im, Ayoung Kang, Soo-Hong Kim, Sang Su Lee, Hyuk Jae Jung

**Affiliations:** a Department of Surgery, Pusan National University Yangsan Hospital, Yangsan, Gyeongsangnam-do, Republic of Korea; b School of Medicine, Pusan National University, Busan, Republic of Korea; c Division of Pediatric Surgery, Department of Surgery, Pusan National University Children’s Hospital, Yangsan, Gyeongsangnam-do, Republic of Korea; d Research Institute for Convergence of Biomedical Science and Technology, Pusan National University Yangsan Hospital, Yangsan, Gyeongsangnam-do, Republic of Korea.

**Keywords:** adolescent, anticoagulation, congenital anomaly, deep vein thrombosis, inferior vena cava

## Abstract

**Rationale:**

Deep vein thrombosis (DVT) is rare in pediatric patients. Congenital inferior vena cava (IVC) anomalies are asymptomatic; however, these can cause DVT in younger patients rarely.

**Patient concerns:**

In this case, a 14-year-old male patient was referred to our hospital for lower abdominal pain.

**Diagnoses:**

At the first hospital he visited, computed tomography revealed a thrombus in the veins of the abdomen and lower extremities; however, an accurate diagnosis could not be made because the thrombus was thought to be inconsistent with abdominal pain. Close examinations revealed swelling of the lower extremities and pain while walking. Contrast-enhanced computed tomography revealed the absence of IVC (AIVC), resulting in DVT at the external iliac, gonadal, inferior mesenteric, and collateral veins of the right pelvic cavity.

**Interventions:**

The patient received anticoagulation therapy.

**Outcomes:**

At a follow-up 6 months later, his symptoms improved, and a thrombus was not found. AIVC could cause DVT.

**Lessons:**

DVT is rare in pediatric patients and is difficult to diagnose when the initial symptoms are vague. Conservative treatments are the most commonly used treatment; long-term follow-up is needed.

## 1. Introduction

Congenital anomalies of the inferior vena cava (IVC) vary because, during gestation, IVC is formed by the complex anastomosis of several veins. Depending on the reports, incidences differed; however, the occurrence rate in the overall population is 0.07% to 8.7%.^[1,2]^ IVC anomalies are asymptomatic in most cases. However, it can cause various serious problems. In cases of infrarenal agenesis of the IVC (AIVC), deep vein thrombosis (DVT) in the lower extremities would occur due to venous stasis.^[2–4]^ If it is not recognized before procedures for vascular, retroperitoneal organ, and kidney transplant surgeries, it can cause major problems or injuries to the vascular systems or organs.^[1,5]^

Here, we discuss a case of AIVC leading to DVT in the lower extremities and pelvic organs in a 14-year-old boy who previously did not have any medical problems, with a brief review of its clinical features, diagnosis, and treatment. This condition is scarce in clinical practice; therefore, it must be reported to aid clinicians’ decision-making regarding diagnosis and treatment.

## 2. Case presentation

A 14-year-old male presented with a 2-week history of right lower quadrant abdominal pain and right leg pain. He was referred to our hospital for thrombophlebitis of an unknown cause. The patient had no risk factors associated with venous thromboembolism, no recent long-distance travel, surgery, trauma, immobilization, or family history of coagulopathies. He was a nonsmoker and had no regular medications. Over the past year, the patient had experienced a rapid growth spurt of approximately 10 cm and was actively engaged inhigh-intensity physical training, including daily basketball practice for several hours.

On physical examination, the whole right leg was swollen. In addition, right lower quadrant and suprapubic tenderness were observed during the abdominal physical examination. Routine laboratory test results were normal except for erythrocyte sedimentation rate (42 mm/h; normal range: 0–10 mm/h) and high-sensitivity C-reactive protein (15.01 mg/dL; normal range: 0–0.5 mg/dL). The prothrombin international normalized ratio and activated partial thromboplastin time were all within the normal range (prothrombin international normalized ratio, 1.14; normal range: 0.97–1.30; activated partial thromboplastin time, 35.8 seconds; normal range: 33.9–46.1 seconds). The antithrombin III activity was normal (109%; normal rage: 80–120%), while the d-dimer was elevated (6.96 µg/mL; normal range: <0.5 µg/mL). Coagulation factors, including clotting factor VIII Ab, protein C Ag, protein C activity, protein S Ag, protein S activity, Lupus anticoagulant screen test, and antiphospholipid antibody, did not have any defects predisposing to thrombophilia. The factor V Leiden mutation was not observed.

**Figure 1.**
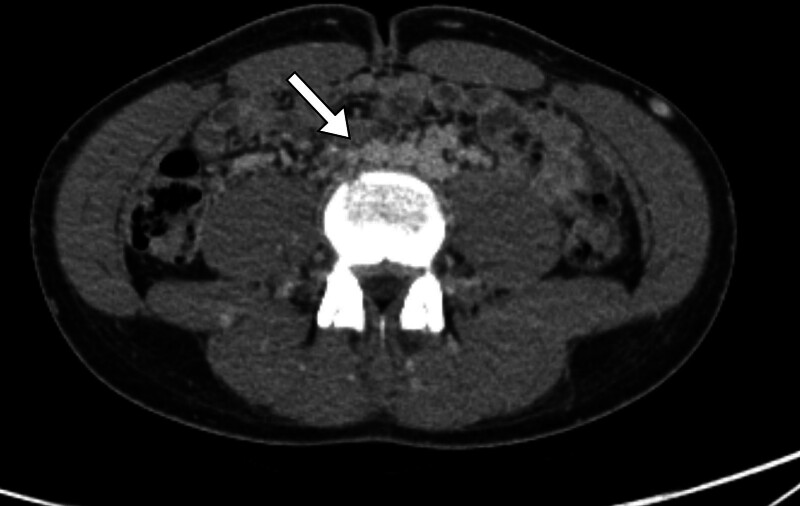
CT image of pre-anticoagulation. The absence of infrarenal IVC. CT = computed tomography, IVC = inferior vena cava.

**Figure 2.**
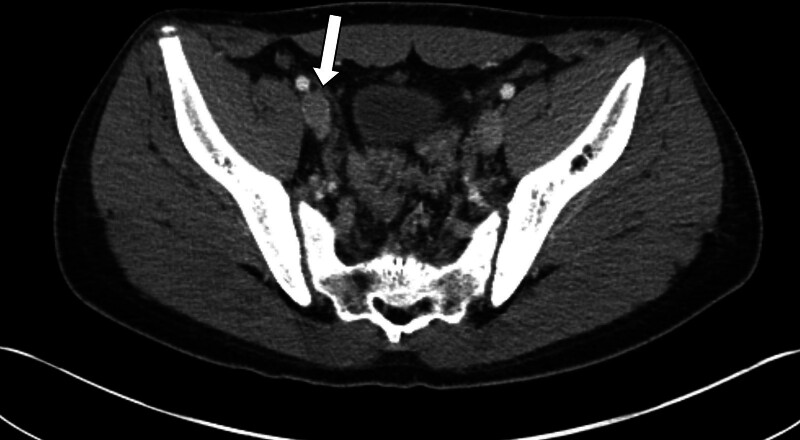
CT image of pre-anticoagulation. Thrombus at right external iliac vein. CT = computed tomography.

Based on the physical examination findings and laboratory results described above, differential diagnoses – including appendicitis, musculoskeletal injury, and deep pelvic infection – were initially considered. Abdominal and pelvic computed tomography (CT) was subsequently performed for further evaluation (Supplementary Video 1). CT revealed the absence of infrarenal IVC (Fig. 1) and bland thrombus at the external iliac, gonadal, inferior mesenteric, and collateral veins of the right pelvic cavity (Fig. 2). No thrombus in the bilateral pulmonary arteries or other anatomical abnormalities were observed. Because contrast-enhanced CT revealed clear evidence of venous thrombosis and the patient showed no signs of arterial insufficiency, invasive angiography was not performed.

As the patient had no other identifiable risk factors for thrombosis, we presumed that the DVT in both legs was due to congenital IVC anomalies. Therefore, the patient was initially managed with subcutaneous low molecular weight heparin therapy (Clexane^Ⓡ^, Sanofi-Aventis Korea [enoxaparin] 6000 U BID) and IV antibiotics (cefoTAXIME^Ⓡ^, Chong Kun Dang Pharmaceutical Corp [cefotaxime] 1 g TID; Trizel^Ⓡ^, JW Pharmaceutical [metronidazole] 500 mg TID) for 10 days. Administration of subcutaneous LMWH was then changed to an oral anticoagulant (Xarelto^Ⓡ^, Bayer HealthCare AG [rivaroxaban] 20 mg QD). The clinical signs and symptoms of thrombosis improved, and the patient was discharged with a follow-up plan with the vascular surgery and pediatric hematology departments. In this case, surgical intervention was considered but ultimately not performed. The patient’s symptoms were limited to right leg swelling without severe venous obstruction, and the presence of AIVC made it difficult to determine a definitive surgical drainage route. Additionally, the patient responded well to anticoagulation therapy, showing sustained improvement without the need for invasive treatment.

**Figure 3.**
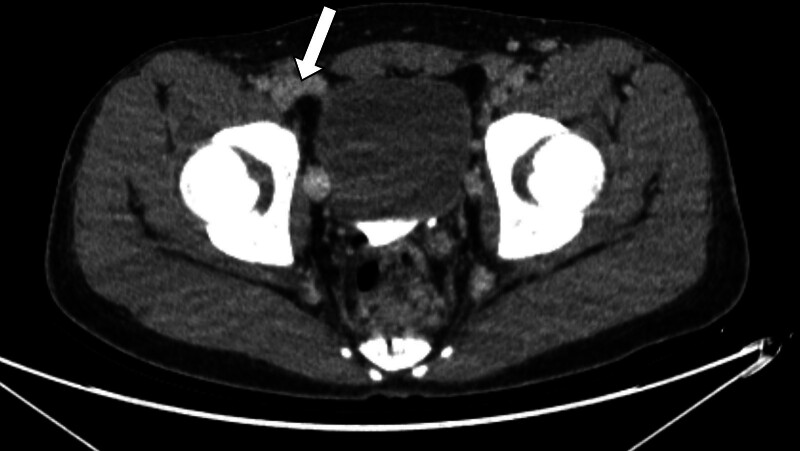
CT image of post-anticoagulation. Right external iliac vein thrombus resolution. CT = computed tomography.

**Figure 4.**
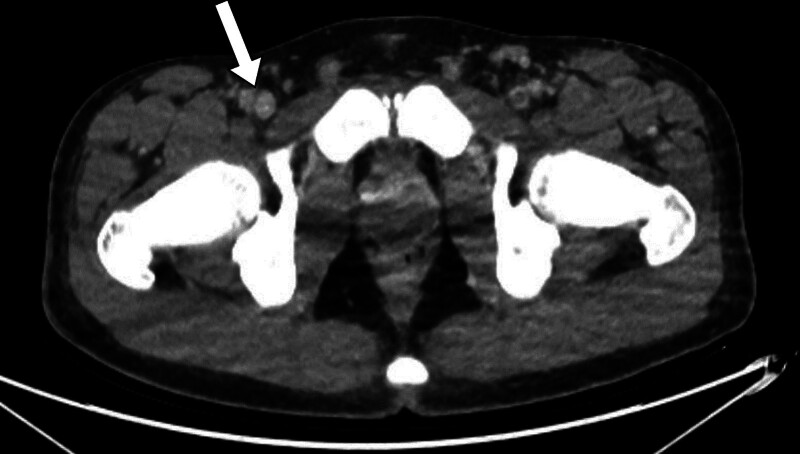
CT image of post-anticoagulation. Right common femoral vein thrombus resolution. CT = computed tomography.

During 2 months of follow-up, the patient had no further symptoms. The administration of Xarelto^Ⓡ^ 20 mg QD was to be maintained for 4 months. After 6 months of oral anticoagulant administration, the patient remained asymptomatic. CT venography revealed a focal thrombus in the right distal external iliac and left common femoral veins, which were otherwise resolved compared with the previous CT (Figs. 3 and 4, Supplementary Video 2).

The patient was advised to avoid excessive physical exertion, prolonged immobilization, and smoking. The use of compression elastic stockings and the intake of oral anticoagulants for life were recommended. Previous studies had reported performing thrombolysis or thrombectomy in addition to anticoagulation. However, in this case, only an oral anticoagulant was administered. No severe symptoms that required invasive interventions, such as thrombolysis or thrombectomy, were observed.

## 3. Discussion

The IVC is developed during embryogenesis, between weeks 4 and 8 of gestation. Additionally, it is a complex event involving the formation, regression, and fusion of 3 pairs of veins: the supracardinal, posterior cardinal, and subcardinal veins. AIVC, like in this case, is a type of vascular anomaly that results from the failure of these veins to fuse. Approximately 90% of congenital AIVC involves the hepatic and suprarenal segments, and only approximately 6% involves the renal or infrarenal segments. Congenital anomalies of the IVC would be frequently associated with various conditions, including cardiovascular defects, situs inversus, polysplenia, asplenia, etc. Approximately 0.3% of the population has congenital IVC anomalies without accompanying cardiac anomalies.^[3,5,6]^

When a deep venous collateral system drains blood from the lower extremities to the heart through the azygos and hemiazygos veins, patients without visceral and heart anomalies are usually asymptomatic. However, like in this patient, developing a collateral deep venous system could be insufficient to cope with the demands of increasing blood flow.^[3]^ In the absence of other provoking factors for DVT, the congenital absence of the infrarenal IVC likely played a major role in the venous stasis and thrombosis observed in this adolescent patient, as reported in similar cases in the literature.^[3,4,7]^

Thromboembolism, such as DVT in children, can have various causes that are different from those in adults. Central venous catheters are the most common contributing factor, and inherited prothrombotic states are important risk factors for venous thromboembolism in pediatric patients. In pediatric patients with venous thromboembolism, it is more common to get abnormal results on thrombophilia and other coagulation testing.^[2]^ DVT due to AIVC with hyperhomocysteinemia has been reported previously.^[7]^ Even if anatomical abnormalities such as AIVC are observed, performing coagulation-related tests is still recommended.

In most cases, DVT associated with IVC anomalies presents with swelling and pain in both lower extremities. However, symptoms that started with lower abdominal or flank pain, although rare, had been reported.^[2–4,8,9]^ In this case, the symptoms began with ambiguous lower abdominal pain, followed by lower extremity pain. At the first hospital the patient visited, though thrombosis was observed on an abdominal CT, its clinical significance was not known because the thromboembolism was not thought to match his symptoms. Most cases of DVT due to AIVC were reported in adult males, and only a few cases were reported in the pediatric population.^[2,4]^ Because DVT is not a common disease in pediatric patients, it would be difficult to diagnose if the symptoms were atypical.

Contrast-enhanced CT or magnetic resonance imaging is useful for diagnosing DVT associated with IVC anomalies because, even when performed to differentiate various diseases without specific impressions, as in this case, these tests are susceptible. Although venography is known as the gold standard for diagnosis, it is highly invasive.^[4]^ For children, it is recommended to use it selectively.

Conservative treatments, including anticoagulation and lifestyle modification, are the most commonly used treatment methods. Wearing compression stockings, quitting smoking, and avoiding long periods of immobilization and excessive physical exertion are recommended. The duration of anticoagulation therapy remains controversial.^[3,4,9,10]^ Surgical and endovascular treatments, such as catheter-directed thrombolysis and thrombectomy, were suggested and reported successful outcomes.^[8,10]^ Long-term and close follow-ups are necessary to monitor recurrences of DVT and bleeding due to anticoagulation.^[8]^ Due to this condition’s rarity, no specific treatment guidelines exist, and further studies are needed.

In summary, AIVC could result in DVT. Because DVT is rare in pediatric patients and unfamiliar to pediatric surgeons and pediatricians, it is difficult to diagnose when the initial symptoms are vague, as in this case. Conservative treatments, including anticoagulation and lifestyle modification, are the most commonly used treatments, and long-term follow-up is needed.

## Author contributions

**Conceptualization:** Dongwon Im, Soo-Hong Kim.

**Supervision:** Sang Su Lee, Hyuk Jae Jung.

**Writing – original draft:** Dongwon Im, Ayoung Kang.

**Writing – review & editing:** Ayoung Kang, Soo-Hong Kim.
